# Finite Element Simulation of Hot Rolling for Large-Scale AISI 430 Ferritic Stainless-Steel Slabs Using Industrial Rolling Schedules—Part 1: Set-Up, Optimization, and Validation of Numerical Model

**DOI:** 10.3390/ma18020383

**Published:** 2025-01-16

**Authors:** Adrián Ojeda-López, Marta Botana-Galvín, Irene Collado-García, Leandro González-Rovira, Francisco Javier Botana

**Affiliations:** 1 Department of Materials Science and Metallurgical Engineering and Inorganic Chemistry, Faculty of Sciences, University of Cadiz, Campus Río San Pedro S/N, 11510 Cadiz, Spain; adrian.ojeda@uca.es (A.O.-L.); leandro.gonzalez@uca.es (L.G.-R.); 2 Titania Ensayos y Proyectos Industriales, Edificio RETSE, Nave 4, Parque Tecnobahía, El Puerto de Santa María, 11500 Cadiz, Spain; marta.botana@titania.aero; 3 Laboratory & Research Section, Technical Department, Acerinox Europa S.A.U., 11379 Palmones, Spain; irene.collado@acerinox.com

**Keywords:** hot rolling, flat rolling, stainless steel, AISI 430, numerical simulation, finite element method

## Abstract

A growing need to reduce the environmental impact and cost of manufacturing stainless steels has led to the development of ferritic stainless steel as an alternative to austenitic and duplex steels. The development of new stainless steels involves the optimization of their hot rolling processes, with the aim of minimizing the occurrence of defects and improving productivity. In this context, numerical simulation using the finite element method (FEM) is presented as a key tool to reduce the time and cost associated with traditional trial-and-error optimization methods. Previous work oriented towards the simulation of stainless steels has been focused on the study of small samples, on the performance of laboratory-scale tests, and on the use of 2D FEM models. In this study, a three-dimensional FEM model is proposed to simulate the hot rolling process of large-scale AISI 430 ferritic stainless-steel slabs using an industrial rolling schedule employed in the actual manufacturing process of flat products. Model optimization is performed in order to reduce the computational cost of the simulations, based on the simulation of the first pass of the hot rolling process of AISI 430 stainless steel. The results show that model optimization reduces the computational time by 90.2% without compromising the accuracy of the results. Thus, it was found that the results for thickness and rolling load showed a good correlation with the experimental values. In addition, the simulations performed allowed for the analysis of the distribution of temperature and effective plastic strain.

## 1. Introduction

In recent years, stainless steel has consolidated its position as one of the most widely used materials in new industrial applications in sectors such as transportation and construction. However, there is a growing interest in introducing new stainless steels with higher added value to the market, with the aim of replacing, in various applications, austenitic and duplex stainless steels, which incorporate nickel [1]. The reduction in nickel consumption would minimize the environmental impact of the stainless-steel manufacturing process [2], making the price of this product more stable and accessible. The manufacturing of sheets of these new steels requires the optimization of rolling processes in order to find processing conditions that minimize the appearance of defects [3].

Flat rolling is one of the most important manufacturing processes in the metallurgical industry. It is estimated that at least 95% of metal alloys are processed at some stage of their productive life by rolling [4,5,6], and about 40–60% of these cases correspond to flat rolling operations [7]. Despite the many advantages that characterize flat rolling, the design and manufacturing of new products by rolling still depend on trial-and-error optimizations, which reduces their efficiency. This is particularly pronounced in hot rolling due to the complexity of the thermo-mechanical metallurgical phenomena involved. Moreover, during rolling, the formed parts are susceptible to unforeseen deformations that complicate optimization [8,9].

To reduce the costs and time spent on the development of new rolling schedules and the optimization of existing ones, several analytical and numerical methods have been used [10,11]. These allow us to analyze the modifications of the conditions of a rolling schedule and the behavior of the material and to create new operations and optimize existing ones. They also contribute to increasing the speed of existing production processes and product quality [12]. Examples include the slab method, the slip-line field method, the upper bound method, the boundary element method, the finite difference method (FDM), and the finite element method (FEM) [7]. Compared to other techniques, finite element simulations are the most widely used due to their accuracy and ability to deal with the increasing complexity of processes and diversity of shapes [13,14].

Numerous publications can be found in the literature using numerical simulation as a tool for the study, development, and optimization of hot rolling processes for stainless-steel flat products. In [15], a review of the state of the art on the numerical simulation of the rolling processes of different metallic materials was performed. McLaren and Sellars [16] combined a 2D FEM model with an FDM model to calculate the mechanics and metallurgy during the hot rolling of 120 mm × 50 mm × 30 mm slabs of 316L austenitic stainless steel. Zhang et al. [17] used a 2D FEM model to investigate the effect of strain accumulation under different strain paths on the static recrystallization behavior of 2 mm thick hot-rolled 304 austenitic stainless-steel strips. Mukhopadhyay et al. [18] developed a 2D FEM model to obtain the local deformation history of the material needed to predict the local microstructural behavior of 100 mm × 30 mm × 18 mm slabs of 316L austenitic stainless steel. Later, in [19], these authors used the same model to study the effect of roll gap geometry on strain behavior during the hot rolling of 316L austenitic stainless-steel slabs of thicknesses less than 50 mm. Sun et al. [20] developed a 2D FEM model to evaluate the effect of rolling parameters on the occurrence of edge seam defects in 304 austenitic stainless-steel slabs 1260 mm in length and 200 mm in thickness when subjected to four hot rolling passes. Mukhopadhyay et al. [21] used a 2D FEM model to calculate the shear strain in hot rolling in two passes of slabs of 316L austenitic stainless steel starting from initial thicknesses of 14.3 mm and 20 mm. Kang et al. [22] employed a 2D FEM model to reveal the effect of lubrication on strain during the three-pass hot rolling of a 10 mm thick strip of ferritic stainless steel with 18 wt% Cr content. Sadiq et al. [23] simulated the hot rolling of small-size flat specimens of AISI 316 austenitic stainless steel with the aim of predicting the values of rolling load and torque. Pourabdollah and Serajzadeh [24] employed an upper bound solution coupled with a two-dimensional thermal FEM analysis to predict the thermo-mechanical behavior of 140 mm × 40 mm × 4 mm AISI 304L austenitic stainless-steel strips subjected to hot and warm rolling. These same authors developed, in [25], a two-dimensional FEM analysis in conjunction with an artificial neural network model to study the microstructural evolutions and mechanical properties of 120 mm × 60 mm × 4 mm AISI 304L austenitic stainless-steel samples when subjected to hot and warm rolling operations. Faini et al. [26] used a 3D FEM model to investigate the impact of hot rolling parameters on the removal of cavity defects in 316L austenitic stainless-steel slabs of 1000 mm × 340 mm × 280 mm after being subjected to a single pass. Rout et al. [27] employed a 3D FEM model to study temperature, strain, and strain rate variations, as well as microstructure differences, in 78 mm × 10 mm × 10 mm specimens of AISI 304LN austenitic stainless steel after being subjected to a hot rolling pass. In [28], Mancini et al. analyzed the origin of edge defects occurring during the hot rolling of 1.4512 ferritic stainless-steel flat products after five roughing passes. Zhou et al. [29] used a 2D FEM model to analyze the temperature and strain distribution in 90 mm thick 436L ferritic stainless-steel slabs during three passes of hot rolling. Wang et al. [30] developed an analytical rolling force model for the hot pack rolling process using the E. Orowan differential equation. The effects of various process parameters were analyzed based on this model, which was validated through a three-dimensional finite element simulation. Simulation was performed using 304 stainless steel for the outer layer and a Ti-Al-2Cr-2Nb alloy for the middle layer. An error margin within 15% was observed between the predictions of the analytical model and the simulated results, demonstrating reliability in predicting rolling force and providing theoretical support for the optimization of the pack rolling process.

Table 1 includes a classification of the publications based on the material, the dimensions of the initial preform, the number of passes, the numerical method used, and the type of discretization applied.

Table 1 reveals that there are few studies in which numerical simulations of the hot rolling process of ferritic stainless-steel flat products have been performed. These investigations primarily focus on 304 and 316 austenitic stainless steels. Austenitic stainless steel is superior to ferritic stainless steel in formability [31]. However, ferritic stainless steels are widely used in various applications due to their excellent thermal conductivity, corrosion resistance, thermal fatigue performance, and relatively low and stable cost [32,33].

Regarding the size of the samples studied, in most cases, small samples were used. Only in [20,26] were large slabs used. Analyzing small samples does not allow for the consideration of phenomena that occur in actual manufacturing processes, such as the appearance of temperature gradients between the different regions of the material.

Additionally, several articles analyze only the first pass of hot rolling. In the rest of the studies, the number of passes is five or fewer. These studies ignore the behavior of the product in more advanced stages of the manufacturing process and how it affects its properties.

When analyzing this table, it can be concluded that all the works found are based on the use of FEM models, which shows that, at present, this type of problem must be studied using this numerical method. Basically, 2D discretization has mainly been carried out, and only in [26,27,30] have 3D models been used. The use of 2D models leads to the simplification of the problem to be solved by assuming that the behavior of the specimen in the width direction is homogeneous. For this reason, the main limitation of 2D FEM simulations is that the effect of edge rolls, the cooling of the material at the edges, or the variation in the strain and stress in these regions is obviated.

In summary, from the bibliographic review carried out, no evidence was found of existing articles focused on the study of the complete hot rolling schedule of large-format ferritic stainless-steel flat products using 3D FEM models. This is due to the high computational cost of this type of simulation. With the aim of contributing to the progress in the use of this type of tool to be able to perform this type of simulation, this article proposes a 3D FEM model of an industrial rolling mill. This model was validated by comparing the results of the first pass of the rolling process with those obtained experimentally. In addition, different optimization strategies of the model were studied to reduce the computational time without affecting the error in the results. The main contribution of this work is that the proposed optimized model will allow us, in the future, to perform simulations of complete rolling schedules, formed by several passes, of ferritic stainless steels and, by extension, of any type of stainless steel adapting the characteristics of the rolling mill used.

## 2. Materials and Methods

### 2.1. Experimental Procedure

The first part of this study consisted of the optimization of the simulation of the hot rolling of AISI 430 ferritic stainless-steel slabs performed in the facilities of Acerinox Europa S.A.U. (Palmones, Spain). The final objective of this rolling process is to produce 1200 mm wide and 3.5 mm thick sheets. The rolling process starts with the preheating stage. In this stage, the slab, which has initial dimensions of 12,000 mm × 1280 mm × 200 mm, is introduced into a walking hearth furnace until a homogeneous temperature of about 1150 °C is reached throughout the volume of the material. Upon leaving the furnace, the descaling stage is carried out, in which the scale generated on the surface of the slab is removed by means of pressurized water jets.

**Figure 1:**
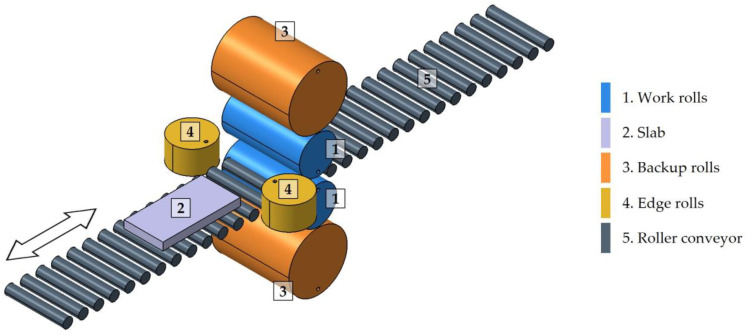
A schematic model of a four high reversible rolling mill used for the roughing stage of the manufacturing of AISI 430 ferritic stainless-steel slabs. The arrow indicates the rolling direction.

Subsequently, the slab is fed into the roughing mill, shown in simplified form in Figure 1. The roughing mill is a reversible quarter mill in which the initial thickness of the 200 mm slab is reduced to a thickness of between 20 mm and 30 mm. This mill has two work rolls, Figure 1(1), between which the material to be rolled passes, Figure 1(2). The backup rolls, Figure 1(3), are larger in diameter, and their role is to transmit the stress to the work rolls. In addition, the mill has two edge rolls, Figure 1(4), which act in specific passes and allow the edges of the rolled product to be of good quality and keep the width of the slab within the desired margins. In addition, the mill has a roller conveyor, Figure 1(5), which transports the slab during rolling. Thickness reduction is achieved progressively by means of a rolling schedule consisting of an odd number of passes, seven being the usual number for this type of product. In passes one to three, a secondary descaling is performed to remove the scale generated in the first passes of the roughing stage.

Table 2 includes basic parameters used in the hot rolling schedule of typical AISI 430 ferritic stainless-steel slabs to obtain sheets with a target width of 1200 mm and a target thickness of 3.5 mm. Specifically, this table provides the values of the initial thickness, the final thickness, the applied reduction ratio, the roll gap, the roll gap of the edge rolls, and the linear speed in each pass.

### 2.2. Material

The material studied was ferritic stainless steel AISI 430/EN 1.4016 quality ACX 490 manufactured by Acerinox Europa S.A.U., whose chemical composition is shown in Table 3.

The hot rolling process of slabs made of the specified material was simulated, with dimensions of 2000 mm in length, 1280 mm in width, and 200 mm in thickness. These dimensions are considered representative of the process conducted on an industrial scale.

### 2.3. FEM Simulation

#### 2.3.1. Establishment of FEM Model

In this paper, a three-dimensional thermo-mechanical analysis of the hot rolling process of AISI 430 ferritic stainless-steel slabs was performed. The modeling and simulation were carried out using the FEM-based software Simufact Forming 2024.2 from Hexagon AB (Stockholm, Sweden). This software is widely used in the numerical analysis of the rolling process due to its capabilities in complicated nonlinear calculation [34]. Specifically, the direct multiprocess solver Pardiso Direct Sparse from Marc^®^ was used, and 4-core parallel computing was employed.

The simulation study was conducted on a workstation with 64 GB RAM and an Intel^®^ Core^TM^ i7-7700K processor with 8 cores and a maximum frequency of 4.50 GHz.

The following assumptions were set:

1. The work rolls, the edge rolls, and the roller conveyor were considered as rigid bodies because of their minimal deformation compared with the slab. Therefore, no material was defined for the rolls.
2. The slab was assumed to be a uniform and isotropic visco-plastic body at the beginning of the simulation.
3. The rolls were assigned a four-node thermally coupled tetrahedral element mesh. In addition, an element type analysis was performed using two different element types for the slab. Specifically, a four-node thermally coupled tetrahedral element mesh and an eight-node thermally coupled hexahedral element mesh were used. A remeshing condition was implemented to avoid mesh distortion caused by deformations occurring during rolling. Thus, the mesh effectively adapted to the changing geometry of the slab, without compromising its integrity or the accuracy of the results obtained.
4. During hot rolling, there is friction between the rolls and the slab. Therefore, the friction coefficient of each pair of contact surfaces was defined.
5. The thermal effects were considered for the thermo-mechanical calculations, including the heat transfer between the slab and rolls and the slab and the environment. Therefore, the heat transfer coefficients were defined. In addition, the temperature of the environment and the temperature of the rolls were also defined.

#### 2.3.2. Model Description

**Figure 2:**
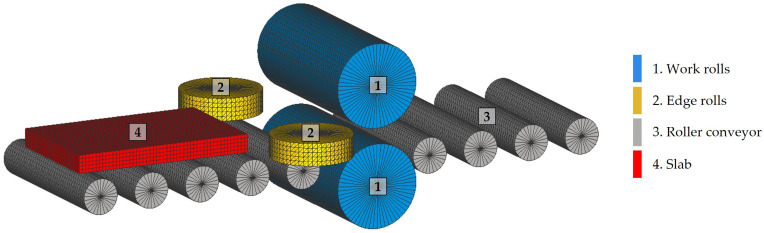
The initial model of the rolling mill used in the finite element simulations of the roughing stage.

The simulations were performed using the initial model shown in Figure 2. This model consisted of two work rolls, Figure 2(1); two edge rolls, Figure 2(2); a nine-roll roller conveyor, Figure 2(3); and the slab, Figure 2(4). The basic rolls and slab parameters used in the simulations are listed in Table 4.

The geometries of the work rolls, edge rolls, roller conveyor, and slab were generated in Blender^®^ 4.1 from the Blender Foundation (Amsterdam, The Netherlands) and exported as STL files, which were subsequently imported into Simufact Forming.

#### 2.3.3. Model Meshing

In this study, a computational model was developed using four-node tetrahedral elements to represent the work rolls, Figure 2(1); the edge rolls, Figure 2(2); and the roller conveyor, Figure 2(3). In order to discretize the rolls, a constant element size of 50 mm was established, achieving an adequate representation of their geometry. As a result, the initial model was discretized in a total of 47,495 elements, which allowed for an adequate resolution to model the main mechanical interactions between the rolls and the slab.

Additionally, an exhaustive element type analysis and mesh sensitivity analysis were performed. These analyses made it possible to identify the type of element best suited to the process conditions and to determine the optimum mesh size to represent the slab, achieving a balance between result accuracy and computational efficiency. In this way, specific meshing criteria were established that improved the capture of deformation and material flow during rolling.

A remeshing condition was implemented to avoid mesh distortion caused by deformations occurring during rolling. Thus, the mesh effectively adapted to the changing geometry of the slab, without compromising its integrity or the accuracy of the results obtained.

#### 2.3.4. The Mathematical Model of the Material

Preliminary simulations of the first pass of hot rolling were performed using the properties of AISI 430 ferritic stainless steel, designated as X6CrTi17_h, whose data sheet can be found in the Simufact Forming materials library. This tab provides the thermo-mechanical properties of the material in a temperature range between 700 °C and 1300 °C, suitable for the simulation of the hot rolling process. Due to the lack of experimental data of the material at the temperatures of interest, thermo-visco-plastic behavior was described using the GMT analytical model, Equation (1). This model allowed us to calculate the flow stress (*σ*_f_) and to guarantee a continuous description of the material behavior during the simulation, considering the strain (φ), the strain rate ($\dot{\varphi }$), and the temperature (*T*). Table 5 provides the material coefficients of Equation (1), which are calibrated for a temperature range between 700 °C and 1300 °C and effective plastic strain of up to 2.

(1) $$\sigma_{f}=c_{1}\cdot e^{c_{2}\cdot T}\cdot \varphi^{n_{1}\cdot T+n_{2}}\cdot e^{\frac{l_{1}\cdot T+l_{2}}{\varphi }}\cdot {\dot{\varphi }}^{m_{1}\cdot T+m_{2}}$$

where

$\sigma_{f}$: flow stress, in N·m^−2^.

φ: strain, dimensionless.

$\dot{\varphi }$: strain rate, in s^−1^.

*T*: temperature, in °C.

*c*_1_: constant depending upon the slab material, in N·m^−2^·s^(m^_1_^T+m^_2_^)^.

*c*_2_: coefficient based on material sensitivity to temperature, in °C.

*n*_1_: coefficient based on temperature and strain coupling, in °C.

*n*_2_: coefficient based on the strain sensitivity of the material, dimensionless.

*l*_1_: coefficient based on temperature and strain coupling, in °C.

*l*_2_: coefficient based on the strain sensitivity of the material, dimensionless.

*m*_1_: coefficient based on temperature and strain rate, in °C.

*m*_2_: coefficient based on strain rate, dimensionless.

#### 2.3.5. Rolling Process Parameters

As mentioned in Section 2.1, the rolling process under study consists of three stages: heating, descaling, and roughing. The heating simulation was carried out by setting an initial temperature of the preforms of 35 °C and an ambient temperature of 1165 °C, typical of this process. The heating time was set at 200 min, deemed sufficient for the slabs to reach the target temperature.

The descaling was simulated at an ambient temperature of 35 °C, a duration of 5 s, and a heat convection coefficient equal to 2000 W·m^−2^·K^−1^ [35].

The first pass of the roughing stage of the hot rolling process was simulated using the key parameters listed in Table 2. A friction coefficient value of 0.25 was assumed. Regarding the thermal conditions, an ambient temperature of 35 °C and an initial temperature of the work rolls of 80 °C were set. A heat convection coefficient equal to 20 W·m^−2^·K^−1^ [14,26] and a heat conduction coefficient between the work rolls and the slab of 15,000 W·m^−2^·K^−1^ [14,24] were defined.

Several modifications of the model were implemented in order to optimize the computing time and minimize the error compared to the experimental results. Finally, the optimized model was used to simulate the first pass of the industrial rolling process. From these simulations, the results of the thickness and load, as well as the temperature distribution and the effective plastic strain, were extracted.

## 3. Results and Discussion

### 3.1. Model Optimization

FEM-based simulations have a high computational cost. The main objective of this part of the work was to develop strategies to optimize the computational time in the numerical simulations of the analyzed process, ensuring that such modifications do not compromise the accuracy and quality of the results obtained. The strategies used and the results obtained are presented below.

#### 3.1.1. Mesh Sensitivity Analysis

The accuracy of an FEM-based analysis depends, among other factors, on the size of the mesh used [36]. Thus, coarse meshes are used in the preliminary stages of simulation processes in order to quickly obtain inaccurate results. In contrast, fine meshes are used to obtain accurate results, with a high computational cost [37]. When working with models consisting of a large number of elements, it is advisable to optimize the mesh size in order to achieve a compromise between the accuracy of the results and the computational cost. For this reason, a mesh sensitivity analysis of the simulation results against the variation in the mesh size of the slab was carried out. This analysis made it possible to determine the value of the mesh size at which the results converge.

**Figure 3:**
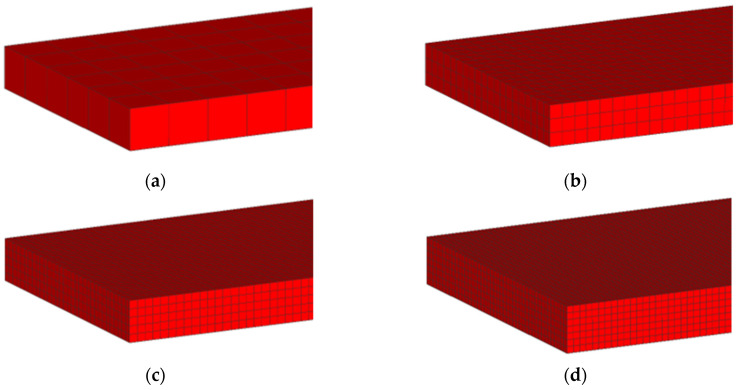
The evolution of the number of element layers in the thickness direction as a function of mesh size: (**a**) 200 mm mesh size and 1 layer; (**b**) 65 mm mesh size and 3 layers; (**c**) 40 mm mesh size and 5 layers; (**d**) 29 mm mesh size and 7 layers.

The analysis was performed by simulating the first pass of the hot rolling process of a 2000 mm × 1280 mm × 200 mm slab, focusing on the rolling load results. The mesh was obtained using eight-node hexahedral elements of variable size, whose values are included in Table 6. According to this table, elements from 200 mm to 29 mm in size were used. Thus, the number of finite element layers present in the thickness direction varies. Figure 3 shows that the number of layers in the model varies from one to seven as the mesh size varies from 200 mm to 29 mm. In addition, Table 6 shows that the number of elements in the slab increases from 60, when the mesh size is 200 mm, to 21,252 for elements 29 mm in size. The purpose of this analysis was to find the optimum number of elements into which the thickness of the slab should be divided. In this way, by varying the thickness of the slab, the number of elements into which the slab thickness is divided will be kept constant by varying the size of the elements.

Table 6 shows the values of the computational time invested in simulating the first pass of the rolling process for each of the seven mesh sizes studied. In addition, the average simulated value of the load is included. It is observed that, as the mesh size decreases, there is an increase in the computation time associated with the increase in the number of elements in the model.

**Figure 4:**
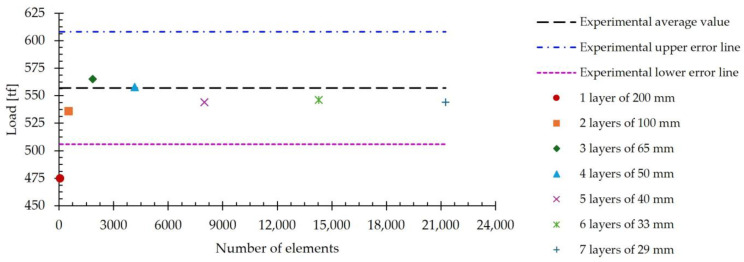
The average simulated rolling load for meshes of different numbers of elements and a number of element layers across the slab thickness ranging from one to seven. A line representing the experimental average value and two upper and lower error lines derived from the standard deviation are included for reference.

Figure 4 shows the average values of the simulated load, included in Table 6, versus the number of element layers across the slab thickness. According to Table 6, seven cases are represented, in which the number of layers varies from one to seven. As a reference, the experimental average load value of 557 tf is included in this figure. Additionally, the upper and lower error lines derived from the standard deviation of the experimental data, which is 51 tf, are also shown. Convergence is considered to be reached when the value of the load does not vary as the number of elements increases. When analyzing the information included in Figure 4, it is observed that convergence is achieved when five layers of 40 mm finite elements are used. By increasing the number of layers above five and, therefore, by reducing the size of the elements below 40 mm, no significant variations in the estimated value of the load are observed.

According to these results, using a mesh size of 40 mm, it is possible to estimate the load with an error percentage of 2.3%. In addition, under these conditions, the five elements into which the thickness of the slab is divided allow the variation in other simulated results to be accurately analyzed as a function of position.

#### 3.1.2. Element Type Analysis

**Figure 5:**
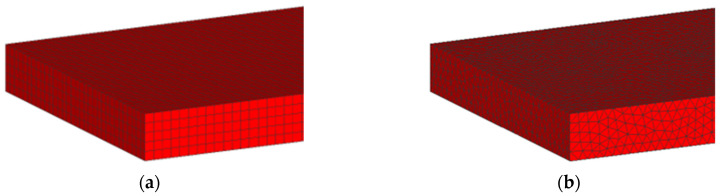
(**a**) Slab mesh using hexahedral elements. (**b**) Slab mesh using tetrahedral elements.

An element type analysis was performed by simulating the first pass of the hot rolling process. For this purpose, a slab mesh composed of eight-node hexahedral elements and another composed of four-node tetrahedral elements were used, Figure 5. An element size of 40 mm was used, which was the optimum value defined in the mesh sensitivity analysis. Thus, the resulting slab mesh consisted of 8000 hexahedral elements, Figure 5a, and 59,700 tetrahedral elements, Figure 5b.

**Figure 6:**
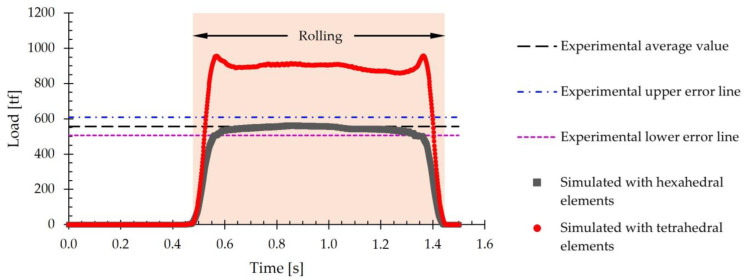
Rolling load during the first pass of hot rolling using a mesh consisting of five layers of hexahedral elements and a mesh of tetrahedral elements. A line representing the experimental average value and two upper and lower error lines derived from the standard deviation are included for reference.

Figure 6 includes the evolution of the simulated rolling load over time using both models. The experimental average load value of 557 tf is included in this figure as a reference. Additionally, the upper and lower error lines derived from the standard deviation of the experimental data, which is 51 tf, are also shown.

Figure 6 shows that when using tetrahedral elements, the rolling load values fall outside the range defined by the experimental error lines. Specifically, the simulated average value for this type of element is 896 tf. In contrast, when using hexahedral elements, the simulated load values lie within the error range and are close to the experimental average value. For hexahedral elements, the average load value is 544 tf. By comparing the average values obtained by simulation with the experimental value, it is concluded that when using the tetrahedral mesh, the error obtained when estimating the load is 60.9%, whereas when using the hexahedral mesh, the error is only 2.3%.

Additionally, computation time was compared. It was observed that, when using hexahedral elements, the time required was 27,723 s, whereas for tetrahedral elements, a total of 33,730 s was consumed. This represents an increase of 21.7% in the computation time when using tetrahedral elements with respect to hexahedral elements.

Based on the results obtained, the subsequent simulations were conducted using a slab mesh made up of 40 mm hexahedral elements.

#### 3.1.3. Symmetry Usage Analysis

**Figure 7:**
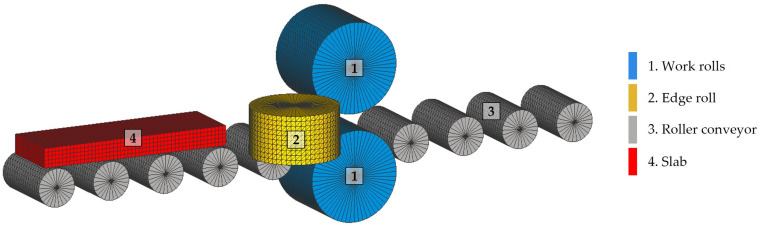
The simplified finite element model of the roughing mill after applying symmetry.

A symmetry usage analysis was performed in order to reduce the computational time. This analysis was carried out by simulating the first pass of the hot rolling process using slabs with a mesh composed of 40 mm hexahedral elements. Figure 7 shows the finite element model of the roughing mill after applying symmetry. In this model, the size of the slab was reduced by half by taking advantage of the existing symmetry of the model with respect to the plane perpendicular to the transversal direction. Thus, the number of finite elements of the slab was reduced from 8000 elements to 4000 elements. Similarly, the length of the work rolls and roller conveyor rolls was reduced to 800 mm. As regards the edge rolls, the left roll was removed. Thus, the number of tetrahedral elements that constituted the rolls was reduced from 47,495 elements to 17,718 elements. In summary, the total number of elements composing the model was reduced from 55,495 elements to 21,718 elements.

According to the information included in Table 6, the time required to simulate the first pass using the original model, formed by 40 mm hexahedral elements, was 27,723 s. By performing the simulation using the symmetry-reduced model of Figure 7, a computation time of 4748 s was obtained. This represents a reduction in computation time of 82.9% compared to the full model.

The effect caused by the simplification of the model was studied by analyzing the rolling load results. As indicated in previous sections, the value of the experimental rolling load was 557 tf, while the average values of the rolling load estimated in the simulations carried out with and without symmetry were 533 tf and 544 tf, respectively. When comparing these simulated values with the value obtained experimentally, it is observed that the error occurring in the estimation increases from 2.3% in the original model to 4.3% in the model reduced by symmetry. Even though the model with symmetry presents a higher error than the model without symmetry, the difference between both values can be considered acceptable, taking into account the savings in computational time obtained by using the simplified model.

**Figure 8:**
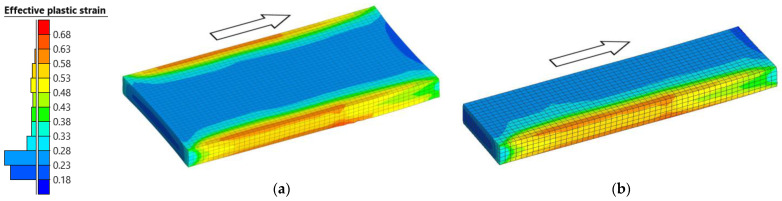
Effective plastic strain distribution at the end of the first pass of the roughing stage. The arrows indicate the rolling direction. (**a**) No symmetry. (**b**) With symmetry.

Additionally, it was analyzed how the use of symmetry affects the strain distribution. Figure 8a shows the distribution of the effective plastic strain at the end of the first pass using the original model, whereas Figure 8b shows the one obtained by using the symmetry-reduced model. As shown in Figure 8a, the effective plastic strain presents a symmetrical distribution with respect to the plane of symmetry. The results obtained using the symmetry-reduced model, Figure 8b, show a similar distribution, with no significant differences.

In summary, the results obtained suggest that the reduction in the size of the model by symmetry considerably reduces the computation time, without causing significant variations in the simulation results. This reduction will be even more significant when simulating the remaining passes of the rolling process, where the size of the elements is considerably reduced. For this reason, the remaining simulations were performed using symmetrically reduced models.

#### 3.1.4. The Simplification of the Model

**Figure 9:**
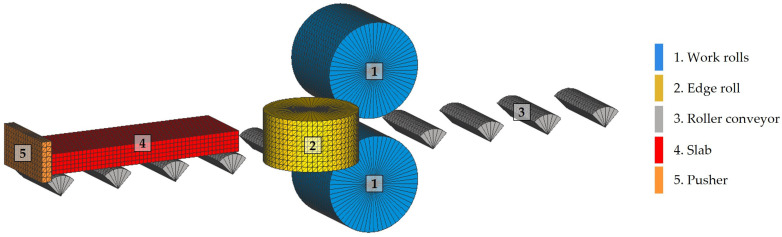
The simplified finite element model of the roughing mill after reducing the size of the roller conveyor and adding a pusher.

In order to further reduce the computational time, an additional modification was made to the model of the roughing mill, which is shown in Figure 9. In this simplified model, the rolls of the roller conveyor were replaced by supports in the form of quarter cylinders. Unlike the rollers used in previous versions of the model, the quarter cylinders are static and serve only a supportive function to counteract the effect of simulated gravity. To replace the movement of the slab on the rolls, a pusher was included, which is responsible for moving the slab during the first moments of the simulation until the bite occurs at the entrance of the rolling mill, at which point slab displacement is produced by the work rolls.

By reducing the size of the roller conveyor rolls, the number of elements in the mesh of the rolls was reduced from 17,718 tetrahedral elements to 13,782 elements. Considering that the slab consists of 4000 hexahedral elements, the complete model was reduced from 21,718 elements to 17,782 elements.

The simulation of the first pass using the model shown in Figure 9 required a computation time of 2727 s, while using the model in Figure 7 required 4748 s. Thus, the simplification introduced in the model shown in Figure 9 reduced the computation time by 42.6%. In turn, an average simulated load value of 538 tf was obtained, which represents an error of 3.4% with respect to the experimental value.

According to the results obtained, the model proposed in this subsection, Figure 9, is functionally equivalent to the model that includes the complete geometry of the roller conveyor rolls, Figure 7. The simplifications introduced in the model allow for a considerable reduction in calculation times without significantly affecting the quality of the simulations performed.

Table 7 summarizes the time reduction obtained by each of the modifications applied to the model. Thus, by using the model described in Figure 9, a 91.9% reduction in computation time was obtained with respect to the complete model based on the use of tetrahedral elements.

### 3.2. Validation of Simulations

According to the results included in the previous section, the different model simplifications proposed allow us to significantly reduce the calculation time without compromising the accuracy of the simulation results. For this reason, the model including all the simplifications was applied to simulate the first pass of the hot rolling process of AISI 430 ferritic stainless-steel slabs. From the simulation, the values of the slab thickness and the rolling load were obtained, which were used to validate the model by comparison with the experimental data provided by the company.

#### 3.2.1. Thickness

**Figure 10:**
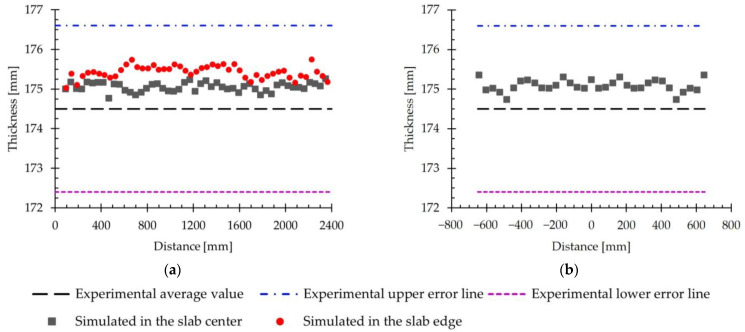
(**a**) The evolution of the simulated thickness in the longitudinal direction measured at the center and at the edge of the slab. (**b**) The evolution of the simulated thickness in the transversal direction measured at the center of the slab. A line representing the experimental average value and two upper and lower error lines derived from the standard deviation are included for reference.

Figure 10 represents the evolution of the slab thickness obtained by simulation, both in the longitudinal direction, Figure 10a, and in the transverse direction, Figure 10b. The thickness values shown in Figure 10a were extracted from both the center and the edge of the slab, whereas in Figure 10b, only values from the center of the slab are included. As a reference, the experimental average thickness value of 174.5 mm is included in this figure. Additionally, the upper and lower error lines derived from the standard deviation of the experimental data, which is 2.1 mm, are also shown.

Both parts of this figure show that the simulated values are within the experimental error lines, specifically between the upper line and the experimental average value. When comparing the thickness results obtained by simulation in the longitudinal direction, Figure 10a, it is observed that the values remain approximately constant, with no major variations in that direction. Likewise, no significant differences are identified between the values recorded at the center and at the edge of the slab. In particular, the calculated average values were 175.1 mm and 175.5 mm, respectively, which were slightly higher than the experimental value of 174.5 mm.

In the analysis of the thickness results in the transversal direction, Figure 10b, a trend similar to that of the longitudinal direction is observed, since there are no significant variations in this direction. The average value obtained was 175.1 mm, coinciding with the average value obtained in the longitudinal direction at the center of the slab.

Extracting thickness values at all slab nodes provides an average value of 175.1 mm, with a standard deviation of 0.2 mm. These results indicate a low variability in the simulated thickness. Table 2 shows that the experimental thickness after the first pass is 174.5 mm, so the error obtained in the simulation is 0.3%.

These results show that the model used is capable of predicting the thickness of slabs processed by hot rolling with precision and accuracy, which makes it a reliable tool for this type of simulation.

#### 3.2.2. Load

**Figure 11:**
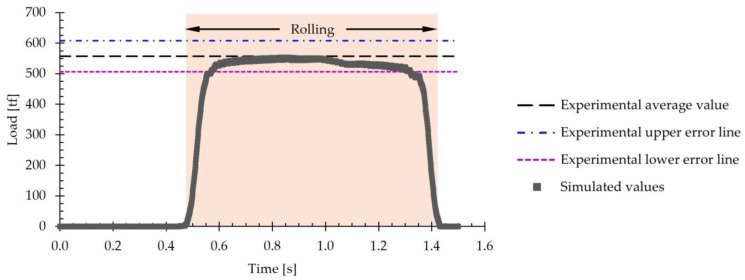
The evolution of rolling load with time for the first pass of hot rolling using the simplified model. A line representing the experimental average value and two upper and lower error lines derived from the standard deviation are included for reference.

Figure 11 presents the evolution of the simulated rolling load over time. This figure includes, as a reference, the experimental average load value of 557 tf. Furthermore, the upper and lower error lines derived from the standard deviation of the experimental data, which is 51 tf, are also shown.

Similarly to Figure 6, the simulated values of the rolling load are between the experimental error lines and close to the experimental average value. An analysis of the evolution of the simulated load shows that it remains practically constant throughout the first pass, with no significant variations over time. The average value obtained in the simulation was 538 tf, and this can be compared to the experimental average value of 557 tf. Comparing both values, it is obtained that the error in the load estimation is 3.4%. In the literature consulted, results with higher errors in the load prediction were considered acceptable. For instance, in [38], rolling loads with errors in the range of ±15% were reported; in [39], errors of up to 14% were recorded; in [40], predictions were achieved with errors of less than 4%; in [41], errors ranged between 10% and 15%; and in [42], errors of up to 24% were reached. The authors attribute these discrepancies to the characteristics of the material model used. Considering these results, the optimized model can be considered to adequately simulate the rolling process studied.

In summary, the results show that the numerical model used is capable of predicting with sufficient precision and accuracy the hot rolling process of large-scale AISI 430 ferritic stainless-steel slabs using industrial rolling schedules. Specifically, the error obtained in the thickness prediction was 0.3%, while that of the rolling load was 3.4%.

The proposed model could be used in the rolling process of other stainless steels by modifying the material properties in the simulation program, it being necessary to validate the results of the simulations by comparison with experimental results.

### 3.3. Simulation Results

In this section, the results of the temperature distribution and the effective plastic strain obtained from the simulations are presented and analyzed. This information could be used to understand the evolution of these magnitudes during the hot rolling process.

#### 3.3.1. Temperature

**Figure 12:**
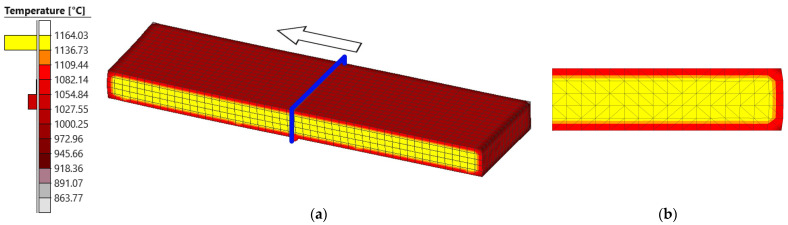
The distribution of temperature obtained at the end of the simulation. (**a**) Half of the slab. The blue plane indicates the cutting plane used to evaluate the temperature in the transversal direction, whereas the arrow indicates the rolling direction. (**b**) A cross-section of the slab.

Figure 12 shows the values of the temperature distribution in the slab at the end of the first pass. The temperature value could not be used in the validation of the simulations, since only the surface temperature value measured at the end of the seventh pass of the rolling process is available. Figure 12a represents the distribution in half of the slab, while Figure 12b shows its distribution in the cross-section obtained by means of a plane perpendicular to the longitudinal direction.

It can be seen in this figure that the temperature distribution in the slab is not uniform, with a thermal gradient between the core and the surface. Thus, it is observed that the temperature is higher in the core of the slab, with values close to the heating temperature in the furnace. When approaching the slab surface, the temperature decreases until an average surface temperature of 1040 °C is reached. It should be noted that the lowest temperatures are reached at the slab edges, this effect being even more pronounced at the corners, Figure 12a, where the temperature drops to 864 °C.

When analyzing the temperature distribution in the cross-sections, Figure 12b, no significant temperature variations are observed in the longitudinal and transversal directions.

**Figure 13:**
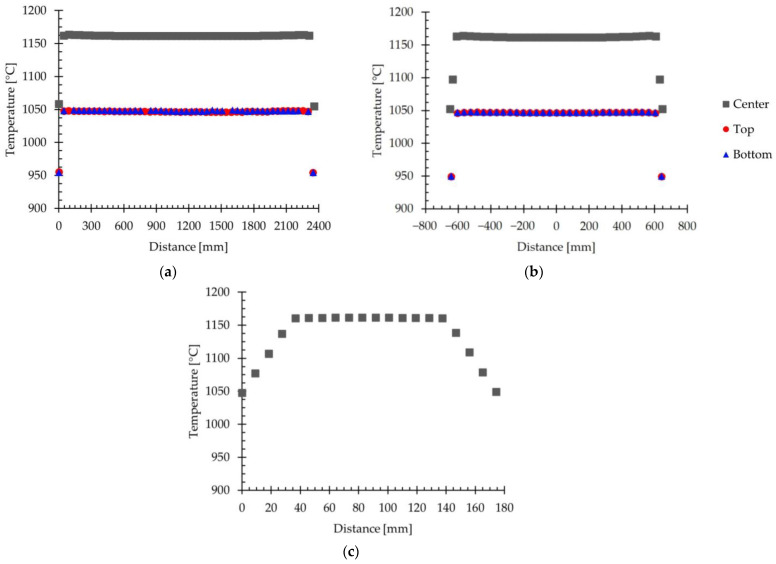
The evolution of the simulated temperature with the position on the slab, evaluated in the center and on the top and bottom surfaces of the slab. (**a**) Evolution in the longitudinal direction. (**b**) Evolution in the transversal direction. (**c**) Evolution in the thickness direction.

In order to study in more detail the temperature variation in the slab, Figure 13a shows the simulated temperature evolution in the longitudinal direction, Figure 13b shows it in the transversal direction, and Figure 13c shows it in the thickness direction. The temperature values represented in these figures were extracted from the center of the slab, as well as on the top and bottom surfaces.

Figure 13a shows that the temperature remains approximately constant along the longitudinal direction in each of the regions evaluated. A decrease in temperature is only detected in the front and tail regions. Furthermore, the difference between the temperature at the center of the slab and at its surface is clearly distinguishable. Specifically, the average temperature at the center is 1162 °C, while at the top and bottom surfaces, it is 1047 °C and 1048 °C, respectively.

The results presented in Figure 13b for the temperature evolution in the transversal direction show a similar trend. In this figure, the temperature remains approximately constant along the width of the slab in the three regions evaluated, presenting only a decrease in the lateral surfaces of the slab. As in Figure 13a, a clear difference is observed between the temperature values in the center and those of the top and bottom surfaces. Thus, the average temperature in the center is 1162 °C, while values of 1047 °C and 1047 °C are obtained on the top and bottom surfaces, respectively.

Figure 13c shows that the temperature evolution in the thickness direction remains practically constant in the core of the slab, with an average value of 1161 °C. However, in the vicinity of the top and bottom surfaces, the temperature decreases to values of 1049 °C and 1047 °C, respectively. This behavior reflects a pronounced thermal gradient near the slab surface.

**Figure 14:**
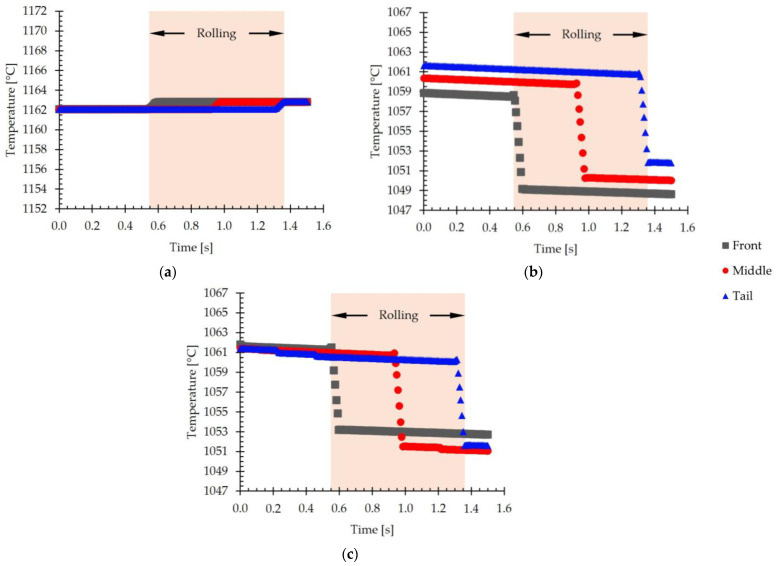
The evolution of the simulated temperature with time evaluated at the front, middle, and tail of the slab. (**a**) The center of the slab. (**b**) Top surface. (**c**) Bottom surface.

Additionally, the evolution of the temperature with the rolling time was analyzed. Figure 14 shows the evolution of the simulated temperature with time evaluated at the front, middle, and tail of the slab for the center, Figure 14a; the top surface, Figure 14b; and the bottom surface, Figure 14c.

Figure 14a shows an increase in the temperature inside the slab when passing between the work rolls, attributable to the heating generated by the plastic deformation, as described in [14,18]. This thermal increase is less than 1 °C. Outside the rolling zone, the temperature inside the slab remains practically constant.

For the top and bottom surfaces of the slab, shown in Figure 14b,c, respectively, a decrease of about 10 °C in temperature during rolling is observed. This phenomenon is attributed to the effect known as “roll chilling”, which results from the thermal transfer between the work rolls and the slab. This thermal gradient, characteristic of the vicinity of the material surface, is due to the high heat conduction coefficient at the roll–slab interface, which generates localized cooling in these regions [14,19].

#### 3.3.2. Effective Plastic Strain

**Figure 15:**
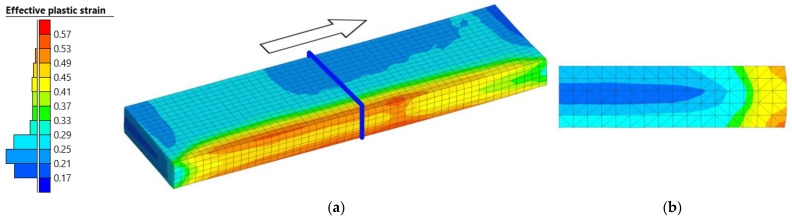
Effective plastic strain distribution obtained at the end of the simulation. (**a**) Half of the slab. The blue plane indicates the cutting plane used to evaluate the temperature in the transversal direction, whereas the arrow indicates the rolling direction. (**b**) A cross-section of the slab.

Effective plastic strain was used to analyze the deformation degree at different positions of the slab at the end of the first pass simulation, as shown in Figure 15. This figure illustrates the results in half of the slab, Figure 15a, and in the cross-section obtained by means of a plane perpendicular to the longitudinal direction, Figure 15b.

As can be seen in Figure 15a, the distribution of the effective plastic strain is not uniform, with gradients in the longitudinal, transversal, and thickness directions. The highest values are obtained, fundamentally, in the lateral edges of the slab, where maximum values of 0.57 are reached. On the contrary, the lowest values are obtained in the center of the slab, where the effective plastic strain is 0.17.

**Figure 16:**
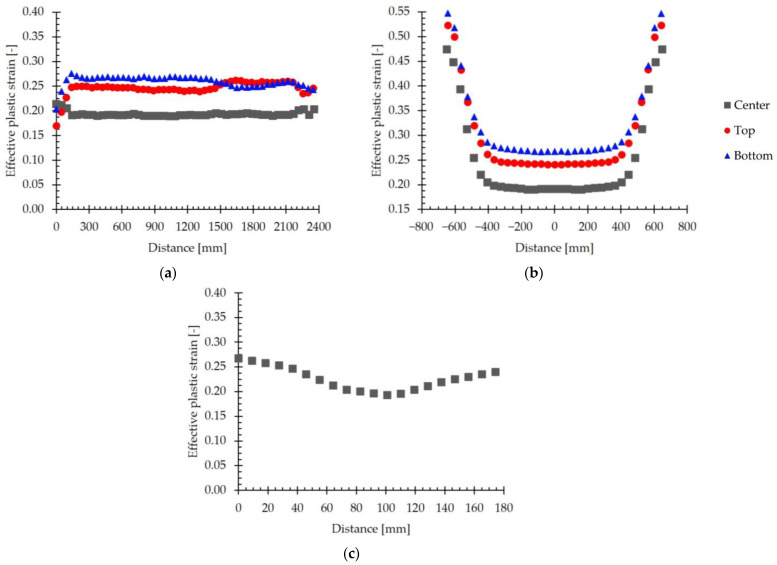
The evolution of the simulated effective plastic strain with the position, evaluated in the center and on the top and bottom surfaces of the slab. (**a**) Evolution in the longitudinal direction. (**b**) Evolution in the transversal direction. (**c**) Evolution in the thickness direction.

To analyze the variation in the effective plastic strain in greater detail, the evolution of the simulated effective plastic strain in different directions is presented in Figure 16. The evolution in the longitudinal direction is depicted in Figure 16a, in the transversal direction in Figure 16b, and in the thickness direction in Figure 16c. The values represented in these figures were extracted in the center of the slab, as well as on the top and bottom surfaces.

Figure 16a reveals that the effective plastic strain remains approximately constant along the longitudinal direction, with no significant differences between the front, center, and tail regions. When comparing the values obtained in the center of the rolled material and on the top and bottom surfaces, it can be seen that the strain is higher on the surface of the material. Specifically, the average values of the effective plastic strain are 0.25 on the top surface, 0.26 on the bottom surface, and 0.19 in the center of the slab.

When analyzing the effective plastic strain in the transverse direction, Figure 16b, it is observed that the strain is lower in the center of the rolled material and that it increases when approaching the lateral edges of the slab. As observed in Figure 16a, the effective plastic strain is greater in the core of the laminated material than on the top and bottom surfaces. Thus, the effective plastic strain at the edges and in the center of the laminated material is, respectively, 0.47 and 0.19 at the core, 0.52 and 0.25 at the top surface, and 0.55 and 0.26 at the bottom surface.

The evolution of the effective plastic strain in the thickness direction, Figure 16c, presents a behavior analogous to that described in Figure 16b for the transverse direction. Thus, the plastic strain reaches a minimum value of 0.19 near the center of the slab, increasing progressively towards the surfaces, where it reaches a value of 0.25 on the top surface and 0.26 on the bottom surface.

These trends coincide with those described in the literature. Thus, in [16], when studying the hot rolling of 316L stainless-steel slabs, it was observed that the calculated strain increases with the distance from the center of the slab due to the presence of redundant shear deformations. In [17], Zhang et al. described the existence of a shear deformation process that tends to concentrate near the surface of the strip when studying the hot rolling process without lubrication of a 304 stainless-steel strip. Xiangyu et al. [43] studied the deformation behavior and bonding properties of a Cu/Al laminated composite plate. The authors observed that the strain obtained on the free surfaces of the Cu/Al plate was higher than that obtained in the core, where the two materials were joined. This phenomenon was also observed by Jiang et al. in [44], where the hot rolling process of a clad plate composed of Q235 carbon steel and 1Cr13 stainless steel was simulated. In this paper, the authors reported that the equivalent strain increases from the core, where the two materials were joined, to the free surface. According to the authors, as the rolling process progresses, the near-surface element deforms more than the core, which is manifested by a greater deformation of the surface metal.

These results reveal that, under conditions of high friction, characteristic of the hot rolling process, there are marked differences in the accumulation of deformation throughout the thickness of hot-rolled products.

## 4. Conclusions

In this study, a numerical model was used to simulate the hot rolling of large-scale AISI 430 ferritic stainless-steel slabs using industrial rolling schedules. An initial model was proposed and modified using different strategies in order to reduce the computational time. Simulations were performed using the commercial finite element software Simufact Forming. Based on the simulations, the values of the thickness of the slab and of the rolling load were obtained. The simulated values were compared with those obtained experimentally in the industrial rolling mill to validate the simulations. The results can be summarized as follows:

- The literature review conducted revealed no previous studies in which simulations of the hot rolling of large format stainless-steel flat products using industrial rolling schedules by means of three-dimensional FEM models have been performed.
- A numerical finite element model was proposed for the simulation of the roughing stage of the hot rolling process of large-scale AISI 430 ferritic stainless-steel slabs. This model was used to simulate the first pass of the rolling schedule used in the industry in the manufacturing of flat products.
- The initial model was enhanced by means of a mesh sensitivity analysis, an element type analysis, the application of symmetry, and a simplification of the model. In this way, the computation time required to simulate the first pass was reduced from the 27,723 s needed to simulate the initial model to the 2727 s used to simulate the improved model. Thus, the computation time was reduced by 90.2%.
- The results obtained indicate that the reduction in computation time does not affect the accuracy of the numerical data, indicating that the model used represents a good compromise between computational time and the accuracy of the results.
- It was estimated that the error obtained in the thickness prediction was 0.3%, whereas the error in the rolling load prediction was 3.4%. These results show that the optimized numerical model can predict with sufficient precision and accuracy the hot rolling process of large-scale AISI 430 ferritic stainless-steel slabs using industrial rolling schedules.
- Results on the distribution of temperature and effective plastic strain were obtained from the simulations performed. Based on the results, it was found that the optimized model is able to correctly represent gradients of these magnitudes, it being possible to appreciate their variations in the longitudinal, transverse, and thickness directions.
- Numerical simulations were conducted for the first roughing pass of steel slabs with a length of 2000 mm. In the case of the studied AISI 430 stainless-steel products, slabs could reach lengths of up to 11,000 mm, which would require significantly longer simulation times. This challenge would become even more pronounced when simulating consecutive passes, where the thickness is substantially reduced. Such reductions necessitate a finer discretization of the finite element mesh to accurately capture the variations in the results along the thickness direction. Future work aims to employ the proposed optimized model to simulate the complete rolling schedule, which consists of seven passes. It is expected that the reduction obtained in the calculation time will allow us to study the complete process in a reasonable timeframe.
- The simulation of the first pass was performed using hexahedral elements. This type of element is well suited to regular geometries, such as the slab, and offers higher accuracy with fewer elements compared to tetrahedral elements. However, meshing complex geometries is more challenging. In industrial hot rolling process, significant deformation is observed at the front and tail of the slab, resulting in the characteristic “fishtailing” effect. These regions exhibit considerable geometric distortions, which can compromise the results of simulations using hexahedral meshes. This aspect should be studied in future work.
- Regarding the material mathematical model, an analytical GMT model was employed. The constants used in this model were calibrated for a temperature range of 700 °C to 1300 °C and an effective plastic strain of up to 2. In the simulation of the first pass, a maximum effective plastic strain value of 0.57 was obtained, suggesting that values exceeding 2 are expected in consecutive passes. Therefore, future studies will need to assess the validity and applicability of the analytical model used.

## Figures and Tables

**Table 1:** The classification of the selected papers based on the material studied, the initial dimensions of the preforms, the number of passes, the numerical method employed, and the type of discretization applied.

Ref.	Material	Length [mm]	Width [mm]	Thickness [mm]	Passes	Numerical Method	Discretization
[16]	AISI 316L	120	50	30	1	FEM; FDM	2D
[17]	AISI 304	N/S	N/S	2	1	FEM	2D
[18]	AISI 316L	100	30	18	1	FEM	2D
[19]	AISI 316L	N/S	N/S	<50	1	FEM	2D
[20]	AISI 304	1260	N/S	200	4	FEM	2D
[21]	AISI 316L	N/S	N/S	14.3; 20	2	FEM	2D
[22]	18%Cr FSS	N/S	N/S	10	3	FEM	2D
[23]	AISI 316	N/S	N/S	N/S	N/S	N/S	N/S
[24]	AISI 304L	140	40	4	1	FEM	2D
[25]	AISI 304L	120	60	4	1	FEM	2D
[26]	AISI 316L	1000	340	280	1	FEM	3D
[27]	AISI 304LN	78	10	10	1	FEM	3D
[28]	1.4512 FSS	N/S	N/S	N/S	5	N/S	N/S
[29]	AISI 436L	N/S	N/S	90	3	FEM	2D
[30]	AISI 304	N/S	N/S	N/S	1	FEM	3D

N/S: not specified; FEM: finite element method; FDM: finite difference method.

**Table 2:** The key parameters of the roughing stage of the hot rolling process. Key data provided by Acerinox Europa S.A.U.

Pass Number	Initial Thickness [mm]	Final Thickness [mm]	Reduction Ratio [%]	Roll Gap [mm]	Edge Roll Gap [mm]	Linear Velocity [m·min^−1^]
1	200.0	174.5	12.8	175.2	1269.0	156
2	174.5	145.6	16.6	147.9	N/A	180
3	145.6	116.2	20.2	116.2	1253.0	196
4	116.2	87.4	24.8	87.0	N/A	214
5	87.4	61.8	29.3	60.7	1253.0	270
6	61.8	41.4	33.0	39.9	N/A	279
7	41.4	27.0	34.8	24.9	1268.0	245

N/A: not applicable.

**Table 3:** Chemical composition of AISI 430/EN 1.4016 ferritic stainless steel (wt.%). Data provided by Acerinox Europa S.A.U.

Fe	Cr	Cu	Mn	Mo	Ni	Si	C	N	P	S
Balance	16.7	0.09	0.37	0.016	0.22	0.41	0.027	0.029	0.022	0.001

**Table 4:** Initial parameters of rolls used in finite element simulations.

Parameters	Values [mm]
Diameter of the work rolls	940
Length of the work rolls	1930
Diameter of the edge rolls	910
Height of the edge rolls	250
Diameter of the rolls of the roller conveyor	400
Length of the rolls of the roller conveyor	1930
Length of the slab	2000
Width of the slab	1280
Thickness of the slab	200

**Table 5:** GMT model parameters for AISI 430 ferritic stainless steel. Extracted from Simufact Materials 2024.2 by Hexagon AB.

*c*_1_	*c*_2_	*n*_1_	*n*_2_	*l*_1_	*l*_2_	*m*_1_	*m*_2_
5862.37 N·m^−2^·s^(m^_1_^T+m^_2_^)^	−4.69 × 10^−3^ °C^−1^	−3.02 × 10^−4^ °C^−1^	3.66 × 10^−1^	−8.78 × 10^−5^ °C^−1^	7.24 × 10^−2^	1.96 × 10^−4^ °C^−1^	−5.47 × 10^−2^

**Table 6:** Meshing data used in mesh sensitivity analysis and convergence study results. Experimental reference load of 557 tf.

No. of Elements in Thickness Direction	Element Size [mm]	No. of Elements of the Slab	Computation Time [s]	Average Load [tf]	Error [%]
1	200	60	7098	475	14.7
2	100	520	7050	536	3.8
3	65	1860	11,063	565	1.4
4	50	4160	15,741	558	0.2
5	40	8000	27,723	544	2.3
6	33	14,274	43,468	546	2.0
7	29	21,252	69,558	544	2.3

**Table 7:** The time reduction achieved by applying different modifications to the model.

Model	No. of Elements of the Slab	No. of Elements of the Mill	Total No. of Elements	Computation Time [s]	Time Reduction [%]
Tetrahedral Elements, Figure 5b	59,700	47,495	107,195	33,730	-
Hexahedral Elements, Figure 5a	8000	47,495	55,495	27,723	17.8
Symmetrical, Figure 7	4000	17,718	21,718	4748	85.9
Simplified, Figure 9	4000	13,782	17,782	2727	91.9

## Data Availability

The original contributions presented in this study are included in the article. Further inquiries can be directed to the corresponding author.
